# The melanopsin-mediated pupil response is reduced in idiopathic hypersomnia with long sleep time

**DOI:** 10.1038/s41598-022-13041-3

**Published:** 2022-05-30

**Authors:** Héloïse Rach, Ulker Kilic-Huck, Eve Reynaud, Laurence Hugueny, Emilie Peiffer, Virginie Roy de Belleplaine, Fanny Fuchs, Patrice Bourgin, Pierre A. Geoffroy

**Affiliations:** 1 Institute for Cellular and Integrative Neurosciences, CNRS UPR 3212 & Strasbourg University, 8 Allée du Général Rouvillois, 67000 Strasbourg, France; 2 CIRCSom (International Research Center for ChronoSomnology) & Sleep Disorders Center, Strasbourg University Hospital, 1 place de l’hôpital, 67000 Strasbourg, France; 3 Inovarion, 251 Rue Saint-Jacques, 75005 Paris, France; 4 Département de Psychiatrie et d’Addictologie, AP-HP, GHU Paris Nord, DMU Neurosciences, Hôpital Bichat-Claude Bernard, 75018 Paris, France; 5 Université de Paris, NeuroDiderot, Inserm, FHU I2D2, 75019 Paris, France

**Keywords:** Sleep disorders, Neuroscience, Circadian rhythms and sleep

## Abstract

Idiopathic hypersomnia (IH), characterized by an excessive day-time sleepiness, a prolonged total sleep time on 24 h and/or a reduced sleep latency, affects 1 in 2000 individuals from the general population. However, IH remains underdiagnosed and inaccurately treated despite colossal social, professional and personal impacts. The pathogenesis of IH is poorly known, but recent works have suggested possible alterations of phototransduction. In this context, to identify biomarkers of IH, we studied the Post-Illumination Pupil Response (PIPR) using a specific pupillometry protocol reflecting the melanopsin-mediated pupil response in IH patients with prolonged total sleep time (TST > 660 min) and in healthy subjects. Twenty-eight patients with IH (women 86%, 25.4 year-old ± 4.9) and 29 controls (women 52%, 27.1 year-old ± 3.9) were included. After correction on baseline pupil diameter, the PIPR was compared between groups and correlated to sociodemographic and sleep parameters. We found that patients with IH had a lower relative PIPR compared to controls (32.6 ± 9.9% vs 38.5 ± 10.2%, p = 0.037) suggesting a reduced melanopsin response. In addition, the PIPR was not correlated to age, chronotype, TST, nor depressive symptoms. The melanopsin-specific PIPR may be an innovative trait marker of IH and the pupillometry might be a promising tool to better characterize hypersomnia.

## Introduction

Hypersomnolence, characterized by an excessive daytime sleepiness (EDS), sleep inertia and/or impaired vigilance^1,2^, is a public health problem affecting about 8.4% of the general population^3,4^. It is the core symptom of central nervous system hypersomnia which includes Idiopathic Hypersomnia (IH), Narcolepsy and Klein-Lewin syndrome. In the latest ICSD-3 definition^1^, IH is characterized by an excessive day-time sleepiness, either a prolonged total sleep time (TST) on 24 h and/or a reduced sleep latency. The prevalence of IH is of 1 in 2000 in general population^4^ and is more common in females^5,6^. IH is underdiagnosed^3^ and often inaccurately treated despite colossal social, professional and personal impacts^7^. This sleep disorder is indeed associated with a decrease in global functioning and an increase in accidents, absenteeism, cardiovascular diseases and mortality^7^. Nevertheless, its pathophysiology remains poorly known. In addition, recent work suggested that abnormalities of the circadian system may contribute to the pathogenesis of IH^8^. IH symptoms are often associated with mood or attention deficit disorders, with hypersomnolence being a core feature of the diagnostic criteria for these disorders^1,2,9–11^. These complex interactions explain the difficulties met by clinicians to distinguish IH from other disorders with, in one hand the shared comorbidity between them and on the other hand the absence of specific biomarkers.

Light is known to exert strong effects on physiology and behavior^12^. These non-visual effects are primarily conveyed by melanopsin-containing intrinsically photosensitive retinal ganglion cells (ipRGCs), which also receive synaptic input from rod-cone networks^13,14^, and provide innervation to various brain areas^15,16^. Light affects sleep and alertness in two ways: (1) indirectly through clock entrainment and phase shifting of circadian rhythms^17^, (2) directly in a circadian independent fashion^18^. It has been recently shown in mice that the non-circadian direct photic regulation of sleep may be mediated by a neural pathway from ipRGCs to the brain preoptic area^19,20^. IpRGCs are also directly involved in the pupillary light reflex^21^, through projections to the olivary pretectal nucleus, and are particularly sensitive to short wavelengths, with a maximal peak at 480 nm corresponding to blue light^22^. These cells have a sustained response after removal of the blue light source which results in continuing constriction of the pupil after light off^23–25^. Interestingly, the amplitude of this prolonged constriction after exposure to blue light, called the Post-Illumination Pupil Response (PIPR), reflects the sensitivity of the melanopsin pathway^26,27^. The PIPR is being increasingly used as a marker of phototransduction in non-visual research on sleep^28,29^, and also for research on alertness and mood^28^. Indeed, it has been recently shown that phototransduction and response to light may be altered in Major Depressive Disorder (MDD)^30,31^ and in Delayed Sleep–Wake Phase Disorder (DSWPD)^29,32^. More specifically, an alteration of the melanopsin response has been suggested in both disorders.

In this context, we hypothesized that the study of melanopsin reactivity to light may help to identify trait-markers of IH. We tested this hypothesis comparing individuals suffering from IH with long sleep time and controls using a pupillometry method that we previously set up, which is highly specific of melanopsin-based phototransduction (PIPR)^23^.

## Methods

### Participants

Participants were men and women aged 18 years or more. Non-inclusion criteria were sleep disorders other than IH (sleep apnea syndrome, circadian rhythm sleep–wake disorders, etc.), day-time sleepiness due to insufficient sleep, other medical conditions known to affect the phototransduction (neurodegenerative disorders like Parkinson disease, Type I and II Diabetes, acquired hereditary retinal disorders), current use of psychostimulant, hypnotic, psychotropic or light therapy, drug or substance abuse, travel across time-zones during the last two months prior to participation, current or past shiftwork, suicidal ideations and pregnancy/breastfeeding. A psychiatric interview (MINI 2.0^33^) was carried out by a trained medical physician to check for current and past mood disorders and substance use.

The control group was recruited through advertisement (online announcement, displays in University and shops). Supplementary inclusion criteria for the control group were no history of sleep disorder (assessed by several questionnaires described in the subjective assessments section) or mood disorders (assessed by the MINI 2.0^33^) and a normal Body Mass Index (BMI) ([18–30]kg/m^2^). Controls also received a thorough eye examination to exclude ocular pathology and a clinical exam.

Patients with confirmed IH were recruited at the Sleep Disorders Center of the University of Strasbourg. The diagnosis protocol includes 3 consecutive days of hospitalization with a continuous video-polysomnography (PSG) recording (detailed below). The first night of PSG assesses the absence of another sleep disorder. On the following day, patients undergo five standard multiple sleep latency tests (MSLT) at 09:00, 11:00, 13:00, 15:00 and 17:00 in order to measure the objective sleepiness (mean sleep latency) and the number of sleep onset rapid eye movement (SOREM) according to validated procedure^34,35^. The continuous recording day starts at the end of MSLT and measures subject’s TST in ad libitum sleep condition.

Diagnostic criteria of IH, in accordance with the International Classification of Sleep Disorders, 3rd edition^1^ are (1) a TST ≥ 660 min (11/24 h) and/or a mean sleep latency during the MSLT ≤ 8 min with no more than one SOREM period, and (2) a subjective EDS ≥ 3 months long evaluated with the Epworth Sleepiness Scale (ESS, detailed below). Only patients with a TST > 660 min were included in this study to correspond to the diagnostic criteria of IH with long sleep time.

The authors assert that all procedures contributing to this work comply with the ethical standards of the relevant national and institutional committees on human experimentation and with the Helsinki Declaration of 1975, as revised in 2008. All participants gave their written informed consent prior to their enrollment in the study (API, 2016 HUS n° 6791) and all procedures have been approved by a French Institutional Review Board on 19 November 2019 (Comité de Protection des Personnes Sud-Ouest and Outre-Mer, number 1-19-070 SI 5025).

### Objective assessments

#### Polysomnography (PSG)

As part of the routine diagnosis protocol, a video-PSG was performed for 3 consecutive days for patients with a suspicion of hypersomnia in chronobiological rooms with controlled conditions: soundproof, temperature of 22 °C ± 0.5 °C, 200 lx white polychromatic light during wake time and darkness during rest time. The monitoring (Siesta, Compumedics) included electroencephalography (C4/M1, C3/M2, F4/M1, F3/M2, O2/M1, O1/M2; 512 Hz, high pass filter = 0.3 Hz; low pass filter = 35 Hz), electrooculography (512 Hz), submental electromyography (250 Hz) and electrocardiography (500 Hz). Scoring was performed in 30 s-epochs with Profusion Software and with a double reading. Tibial electromyograms, thoracic and abdominal belts, nasal cannula, thermistor pulse oxymeter and electrocardiogram were installed to monitor apnea–hypopnea index (AHI), arousal and periodic limb movements during both nights. Patients underwent five standard MSLTs to measure the objective sleepiness and the number of SOREM. Then, the TST was determined during the 24 h monitoring protocol following the first night of PSG habituation and the percentages of non-rapid eye movements (NREM) and rapid-eye movements (REM) sleeps were calculated during both nights. Thus, only the parameters of the 24 h monitoring protocol and MSLT parameters were used for the study. Sleep stages, respiratory events, arousals and periodic legs movements were determined according to standard criteria of the American Academy of the Sleep Medicine (AASM) Version 2.4.

#### Pupillometry

Pupillometry was conducted to assess the melanopsin-mediated pupil response with a protocol published by van der Meijden et al., demonstrating high test–retest reliability^23^. Participants seated in front of the pupilometer (Dioptron®, TOP CON RAM-A2000) with the right eye placed at 5 cm of a light box integrated in the set-up in front of this eye (16 × 10 cm; light source = RGB-LED, Lamina®, NT-43F0-09424 LED, Atlas, RGB, Farnell, Leeds, United Kingdom). Left eye fixed a central target also integrated in the set-up in front of them (Dim green LED) and the pupil diameter variations of this eye was measured with an infrared camera (wavelength = 880 nm) and recorded on a computer at a frequency of 25 Hz. An examiner (HR) was present in the room to adjust the focus and the contrast of the pupil image obtained during the exam but was not allowed to communicate with the patient. Data were retrieved using R software (R version 4.0.5, 2021-03-31, R Foundation for Statistical Computing, Vienna, Austria).

**Figure 1:**
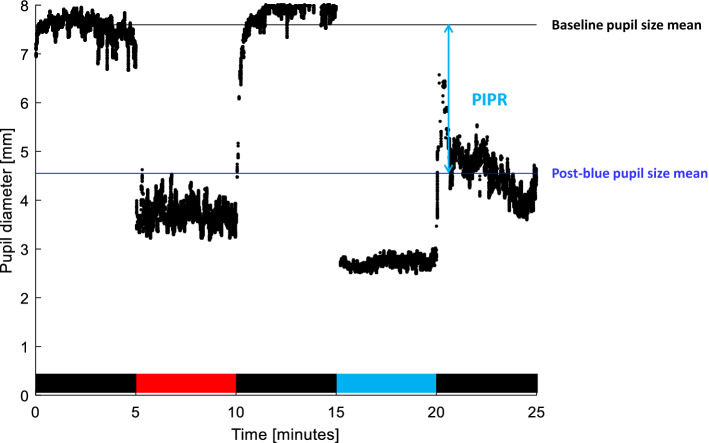
Example of pupil diameter change during pupillometry protocol. The post-illumination pupil response represents the difference of diameter between the first period of darkness and the post-blue darkness (PIPR)^23^. For each block of 5-min, the pupil diameter was averaged on the three central minutes. The absolute PIPR (1) and the relative PIPR (2) were calculated as follows: (1) Absolute PIPR (mm) = (averaged baseline pupil diameter) − (averaged post-blue light exposure pupil diameter); (2) Relative PIPR (%) = 100*(PIPRmm)/averaged baseline pupil diameter. The bottom bar indicates the light exposure sequence: black: darkness, red: monochromatic red light (luminance: 375 cd/m^2^, irradiance: 14.75 log photons/cm^2^/s) and blue: monochromatic blue light (luminance: 375 cd/m^2^, irradiance: 14.57 log photons/cm^2^/s).

The pupillometry protocol was conducted between 9:30 and 16:30 to avoid pupil circadian variations as reported by Zele et al.^36^ and after an adaptation of 30 min to room white polychromatic light of 200 lx. This exam was conducted during the first day of hospitalization for patients with a hypersomnia suspicion or on another day for patients with previously confirmed hypersomnia and for controls. The 25 min standardized protocol^23^ included: 5 min of baseline darkness, 5 min of monochromatic red light (wavelength = 630 nm), 5 min of post-red darkness, 5 min of monochromatic blue light (wavelength = 470 nm) and 5 min of post-blue darkness (Fig. 1). Luminance was the same for all light exposures (375 cd/m^2^), and the corneal irradiance was equal to 14.57 log photons/cm^2^/s for the blue light and 14.75 log photons/cm^2^/s for the red light. The monochromatic red light allowed to maximize the effect of the subsequent blue light in order to obtain a maximum activation of the ipRGCs in the post-blue darkness^23,37^. Five minutes of blue light exposure were used in order to obtain a complete light adaptation of ipRGCs^38^.

The main evaluation criterion of this study was the PIPR, defined as the difference between baseline pupil diameter and the post-blue pupil diameter change of the baseline, which quantifies the functionality of the ipRGCs^26,27^. Absolute PIPR in mm (1), relative PIPR from baseline (in percent) (2), and baseline pupil diameter (3) were calculated^27,39,40^. As previously published^23^, the first and last minutes of 5-min blocks were excluded from analyses in order to reduce the potential interaction between image-forming vision and non-image forming vision photoreceptors after light off and the potential re-dilatation of the pupil in the last minute of post-blue light exposure^13,26^. The parameters used for the subsequent analyses were:

1. Absolute PIPR (mm) = (baseline pupil diameter) − (post-blue pupil diameter)
2. Relative PIPR (%) = 100 × (PIPRmm)/baseline pupil diameter
3. Baseline pupil diameter (mm) = mean of the 3 central minutes of basal darkness

### Subjective assessments

We used the following scales to assess chronotype, sleep/sleepiness and depression self-assessments.

#### Chronotype

The chronotype was assessed with the Morningness Eveningness Questionnaire (MEQ)^41,42^. The total score (TS) was obtained with the sum of the 19 items (TS ≥ 70: definitely morning type; 59 ≤ TS < 70: moderately morning type; 42 ≤ TS < 59: neutral chronotype; 31 ≤ TS < 42: moderately evening type; TS < 31: definitely evening type).

#### Sleepiness, sleep quality and fatigue

The daytime sleepiness was assessed with the Epworth Sleepiness Scale (ESS, TS > 10 on the 8 items: daytime sleepiness)^43,44^ and the sleep quality was estimated with the Pittsburgh Sleep Quality Index (PSQI)^45,46^. The PSQI questionnaire includes 19 items generating 7 component scores: duration of sleep, sleep disturbance, sleep latency, daytime dysfunction due to sleepiness, sleep efficiency, subjective sleep quality, use of sleeping medication (TS > 5: poor sleep quality). The fatigue was evaluated with the Pichot questionnaire (TS > 22 on the 8 items: excessive fatigue)^47^.

#### Depression

Depressive symptoms severity was determined with the Structured Interview Guide for the Hamilton Depression scale—Seasonal Affective Disorder version—Self Assessment (SIGH-SAD-SA). The SIGH-SAD-SA comprises 17 Hamilton Rating Scale items for typical depression (part A) and an additional set of 8 atypical symptoms items (part B). The TS was calculated by adding scores of parts A and B and interpreted as mild (5 ≤ score < 11), moderate (11 ≤ score < 20) or significant (score ≥ 20) level of depressive symptoms severity^48^.

#### Light sensitivity

Subjective light sensitivity was determined with a 10 cm visual analogue scale (VAS) and scored from 0 (no light sensitivity) to 100 (maximal light discomfort).

### Statistical analysis

We first investigated the association between melanopsin-mediated pupil response, sociodemographic and sleep characteristics. We thus computed linear regressions between the relative PIPR (%) (dependent variable) and separately age, sex, chronotype, depressive symptoms, time of the year of testing, TST/24 h, mean sleep latency (MSLT) and subjective sleepiness (ESS) in the whole population and in each group (IH group and controls group). Then, group was included as an interaction factor to assess if the association between pupillometry measures and clinical parameters differed according to diagnosis. We then compared IH patients and controls regarding pupillometry, clinical and sociodemographic parameters using a binomial logistic regression (with group as dependent factor). Pupillometry analysis were adjusted on age, as previous studies have reported an age difference on melanopsin-mediated pupil response^49^. Linear regression was also performed to assess the association between subjective sleepiness (ESS score) and subjective light sensitivity (VAS score) in both groups. Statistical analysis were conducted using the R software (R version 4.0.5, 2021-03-31, R Foundation for Statistical Computing, Vienna, Austria). Statistical significance was set at p < 0.05 for all tests.

## Results

### Clinical and sociodemographic characteristics

Table 1 presents the clinical and sociodemographic characteristics of the participants. Analysis compared 29 controls (mean age 27.1 ± 3.9 years, 52% (N = 15) of women) and 28 patients with IH (mean age 25.4 ± 4.9 years, 86% (N = 24) of women. Groups did not differ in age distribution (p = 0.16) but the prevalence of women was higher in the IH group (p = 0.013), which is in line with the sex-ratio reported in this sleep disorder^50^. The BMI did not differ between groups (p = 0.33).

**Table 1:** Clinical and sociodemographic characteristics of 28 IH patients with Long Sleep Time and 29 Controls.

	IH patients N (%) or mean ± SD	Controls N (%) or mean ± SD	p-value
N	28	29	
Sex (women)	24 (85.71%)	15 (51.72%)	0.008**
Age (years)	25.42 ± 4.92	27.09 ± 3.92	0.163
BMI	22.47 ± 3.85	21.60 ± 2.59	0.33
Chronotype (MEQ)	46.57 ± 11.14	54.72 ± 10.36	0.009**
Epworth sleepiness (ESS)	13.07 ± 3.69	6.59 ± 3.50	< 0.0001***
Pichot fatigue score	15.61 ± 7.13	3.52 ± 2.99	< 0.0001***
Sleep quality (PSQI)	5.35 ± 2.54	2.34 ± 1.56	< 0.0001***
Severity of depressive symptoms score (SIGH-SAD-SA)	12.25 ± 7.76	5.14 ± 4.53	< 0.0001***
Subjective light sensitivity (VAS)	58.68 ± 25.86	53.69 ± 25.92	0.463

*IH* hypersomniacs patients with long sleep time, *Controls* healthy subjects, *BMI* body mass index, *MEQ* Morningness Eveningness Questionnaire, *ESS* Epworth Sleepiness Scale, *PSQI* Pittsburgh Sleep Quality Index, *SIGH–SAD–SA* Structured Interview Guide for the Hamilton depression scale–Seasonal Affective Disorder version–Self Assessment, *VAS* Visual Analogue Scale, *N* size, *SD* Standard Deviation.

p-values are from logistic regression tests. **p-value < 0.01; ***p-value < 0.001.

As expected, patients with IH had higher subjective sleepiness scores (ESS), higher fatigue scores (Pichot questionnaire) and lower sleep quality (higher score obtained at the PSQI) than controls (p < 0.001, Table 1). No differences in subjective light sensitivity were observed. Using the SIGH-SAD-SA rating scale, IH patients showed higher self-rated depression symptoms, with a moderate level of depression (p < 0.001). Although both groups presented neutral chronotype at the MEQ, participants in the IH group had lower scores indicating a later chronotype (p = 0.009).

### Polysomnographic characteristics of patients with IH

IH patients had a prolonged TST on 24 h (746.8 ± 65.02 min) and a mean sleep latency on the MSLT of 12.13 min (± 4.32). Periodic limb movement’s index/hour (7.16 ± 9.20) and AHI/hour (2.81 ± 3.83) were normal and the microwake index/hour was low (15.56 ± 9.76). The polysomnographic recording confirmed the diagnostic of IH with prolonged sleep time and the absence of other sleep comorbidities.

### Relation between PIPR and sociodemographic/sleep characteristics

Age, sex, time of testing, chronotype score, depression score, sleepiness score, were not significantly correlated with the relative PIPR in the whole population (IH and healthy subjects, n = 57), neither in each group (Table 2, all p’s > 0.05) or in the whole population with the diagnostic group included as interaction factor (Table 2, all p’s > 0.05). In addition, mean sleep latency (MSLT) and TST/24 h were not significantly correlated with the relative PIPR in the group of patients with IH.

**Table 2:** Linear regressions between relative PIPR and sociodemographic/sleep characteristics for all volunteers, and for each diagnostic group (29 Controls and 28 IH with Long Sleep Time).

	All population		Controls		IH patients	
	β (SE)	p-value	β (SE)	p-value	β (SE)	p-value
**Sociodemographic characteristics**						
N	57		29		28	
Age	0.074 (0.31)	0.812	− 0.113 (0.49)	0.821	− 0.014 (0.39)	0.972
Sex	3.145 (2.97)	0.294	1.387 (3.83)	0.72	− 0.038 (5.49)	0.994
Chronotype score	0.149 (0.12)	0.223	0.091 (0.18)	0.635	0.038 (0.17)	0.827
Depression score	− 0.272 (0.19)	0.16	− 0.161 (0.43)	0.71	− 0.061 (0.25)	0.809
Time of test	− 0.576 (0.86)	0.503	− 1.809 (1.17)	0.134	− 0.606 (1.26)	0.635
Subjective light sensitivity (VAS)	− 0.013 (0.05)	0.808	0.031 (0.07)	0.683	− 0.036 (0.07)	0.634
**Sleep characteristics**						
Epworth score	− 0.561 (0.28)	0.051	0.385 (0.55)	0.493	− 0.862 (0.50)	0.098
TST/24 h	–	–	–	–	− 0.026 (0.03)	0.385
Mean sleep latency (MSLT)	–	–	–	–	− 0.008 (0.51)	0.987

Linear regressions were realized in the whole population (57 volunteers) and in each group (control group and IH group). No covariate was included in the analysis.

*IH* idiopathic hypersomnia patients with long sleep time, *Controls* healthy subjects, *Relative PIPR* post-illumination pupil response in percent, *VAS* Visual Analogue Scale, *TST* total sleep time, *MSLT* multiple sleep latency test, *β (SE)* linear regression coefficient (Standard Error).

### PIPR comparisons

**Figure 2:**
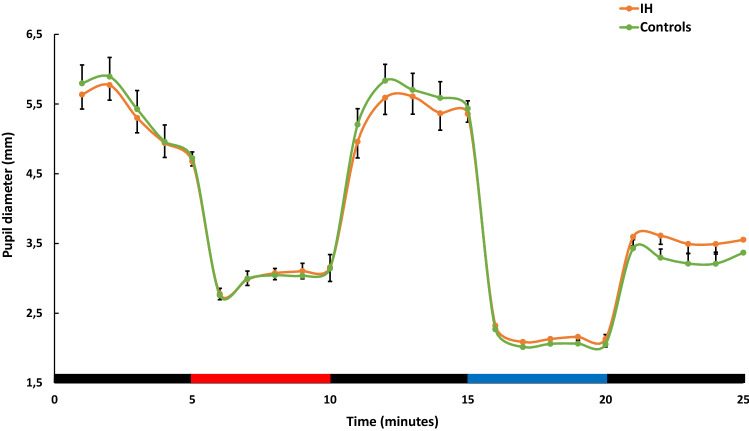
Pupil diameter throughout the pupillometry protocol in patients with IH and control subjects. Each point represents the mean pupil size in mm per minute ± Standard Error Mean. The bottom bar indicates the light exposure sequence: black: darkness, red: monochromatic red light (luminance: 375 cd/m^2^, irradiance: 14.75 log photons/cm^2^/s) and blue: monochromatic blue light (luminance: 375 cd/m^2^, irradiance: 14.57 log photons/cm^2^/s); IH: patients with idiopathic hypersomnia; Controls: healthy subjects.

Table 3 presents the comparisons of pupillometry parameters between groups with age included as a covariate in the analysis (Table 3). When included as a covariate in the analysis, no age difference was found between groups for each pupillometry parameter. Patients with IH showed a significantly lower relative PIPR from baseline pupil diameter in response to blue light than the control group (p = 0.037, model controlled for age). No significant differences were observed between groups for absolute PIPR, nor for the baseline pupil diameter (Fig. 2), although results regarding absolute PIPR were close to those of the relative PIPR (p = 0.071, model controlled for age). In addition, no significant differences were observed between groups for the pupil diameter during blue light exposure.

**Table 3:** Binomial logistic regressions between diagnostic group and pupillary response in 28 IH patients with Long Sleep Time and 29 Controls.

	IH patients	Controls	β (SE)	p-value
	Mean ± SD	Mean ± SD		
Relative PIPR (%)	32.59 ± 9.98	38.54 ± 10.16	**− 0.063 (0.03)**	0.037*
Absolute PIPR (mm)	1.78 ± 0.75	2.17 ± 0.97	**− **0.603 (0.33)	0.071
Baseline (mm)	5.31 ± 1.14	5.31 ± 1.42	**− **0.117 (0.21)	0.588
Blue (mm)	2.14 ± 0.24	2.06 ± 0.29	0.806 (1.06)	0.446

The logistic regression compares each pupillometry parameter between groups (group as dependent factor, controls as reference category), with age included as covariate.

Significant values are in [bold].

*IH* idiopathic hypersomnia patients with long sleep time, *Controls* healthy subjects, *PIPR* post-illumination pupil response, *Baseline* baseline pupil diameter, *Blue* pupil diameter during blue light exposure, *β (SE)* binomial logistic regression coefficient (Standard Error).

Significant logistic regression between the markers: *significant difference between groups, p-value < 0.05.

### Relation between subjective light sensitivity and sleepiness

**Figure 3:**
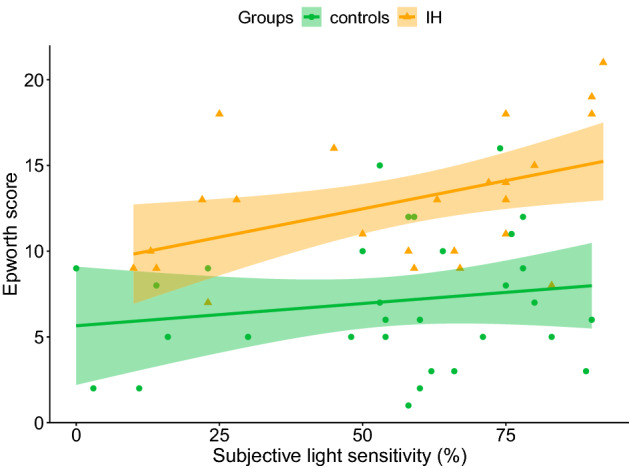
Linear regression between subjective sleepiness and subjective light sensitivity in patients with IH and control subjects. The sleepiness score was obtained using the Epworth scale (ESS) and the subjective light sensitivity was assessed using a Visual Analogue Scale (VAS). The line indicates the association between ESS score and subjective light sensitivity (IH: β = 0.07 ± 0.03, p-value = 0.011; control subjects: β = 0.015 ± 0.03, p = 0.553); IH: patients with idiopathic hypersomnia; Controls: healthy subjects; β: linear regression coefficient.

The association between sleepiness (obtained on ESS) and light sensitivity deferred according to the diagnostic group (p of interaction < 0.001), with a positive regression between subjective sleepiness and light sensitivity in patients with IH (β = 0.07 ± 0.03, p = 0.011; see Fig. 3) while no association was found in controls (β = 0.015 ± 0.03, p = 0.553). In other words, sleepiness significantly increased with light sensitivity only in patients with IH.

## Discussion

The present study characterized the melanopsin response of patients affected by IH with long sleep time, using a pupillometry method. To our knowledge, this is the first study to compare the PIPR between a population of patients with IH and healthy subjects. The relative PIPR was significantly lower in individuals with IH as compared to controls, indicating a lower melanopsin response associated with IH. Interestingly, this finding was not explained by other possible confounding factors as the relative PIPR was not correlated to age, sex, chronotype, time of test, scores of depression and sleepiness in both groups, nor to the mean sleep latency and TST/24 h in IH group. These results comply with the possibility that the relative PIPR could be a trait marker of IH.

Comparison of our results to the literature presents some difficulties, because a multitude of pupillometry protocols and outcome parameters are currently used to assess the PIPR. Thus, discrepancies could be explained by the heterogeneity of methods. A majority of studies using pupillometry assessed the pupil response 6 s after the offset of a blue light flash of around 1 s^30,31,51^ and varying length of dark adaptation before protocol. These protocols allow for a primarily melanopsin-driven response but do not maximize it. Indeed, studies which have investigated the reactivity to blue light in healthy subjects described an extension over time of the pupil constriction in the darkness after longer exposure corresponding to slow response kinetic of the melanopsin intrinsic photoresponse^26,37,52^, a characteristic of the melanopsin response that was used to develop the pupillometry method used in our study^23^. Thus, the present protocol of five periods of 5-min alternating darkness, red and blue exposures conducting to a completed light adaptation of ipRGCs^38^, was used to assess PIPR. In this study, we used the relative PIPR as recommended by Kelbsch et al.^40^ and also reported the absolute PIPR to be able to compare our results with other studies.

The reduced PIPR obtained here shows a less sustained pupil constriction amplitude after exposure to blue light in IH patients compared to healthy subjects. Similar results have been found in MDD^30,31^ and DSWPD^32^. This result is interesting as MDD and IH share common symptoms of hypersomnolence^1,2^. In our study, the relative PIPR was not correlated with depressive symptoms. Although depression score in IH patients was higher as compared to controls, most IH patients did not meet cut-off criteria for a depressive episode. In this context of relatively low depressive symptoms, our recruited population might not allow us to observe an association between PIPR and depressive symptoms. Similarly, participants of the study were young adults and did not present extreme chronotype (morning or evening). The homogeneity of these factors in our population is likely to explain their lack of correlation with the relative PIPR, in discrepancy with previous studies^29,49^. In addition, an objective measure of the chronotype, with the Dim Light Melatonin Onset (DLMO) or the core body temperature, may be more appropriate to address the question of a circadian system abnormalities in IH^8^. Although the absolute PIPR was not significantly different between groups, we observed a tendency to a lower PIPR in IH group. Since the absolute PIPR is not corrected for baseline pupil diameter, the measure is considered as less robust than the relative PIPR and has higher relative variability, which is likely to explain the divergence with relative PIPR.

Additionally, the baseline pupil diameter was not different between groups. The baseline pupil diameter has been poorly studied in IH, but this result is in line with most studies carried out in patients with type 1 narcolepsy, which is currently the best characterized central hypersomnia^53–56^, although contrasting with one older study^57^. It is now well known that the study of the pupillary light reflex is useful to discover impairments of the autonomic nervous system^58^. Indeed, pupil constriction is mediated by parasympathetic nervous system while pupil dilatation by the sympathetic one^59^. A reduced pupil diameter, as found by Pressman et al.^57^, could suggest an increased parasympathetic activity, which is in line with one physiological hypothesis concerning IH^60^, but not found in the current study.

Finally, the subjective light sensitivity (VAS) was positively correlated with the ESS sleepiness score in IH patients meaning that in IH with long sleep time, higher subjective light sensitivity increases with sleepiness.

Globally, these results suggest a dysfunction of the melanopsin system in individuals with IH compared to controls. The observed melanopsin dysfunction in IH could, in part, induce the sleepiness symptoms. Indeed, light exerts a direct influence on sleep and waking, stimulating alertness and inhibiting sleep in humans^14,16,61–63^. A lower melanopsin sensitivity could have the same impact than reduced daylight exposure and lead to a reduced daily alertness and an increased night sleep duration as found in our population of IH patients^64–66^. It has been recently discovered that ipRGCs innervate a part of the brain preoptic area (POA) that induces NREM sleep in mice and whose neurons inhibit wakefulness-promoting brain regions^18,20,67^. In addition, another study showed that POA and tuberomammillary nucleus, structure involved in wake regulation, could operate together as a flip–flop switch that can generate transitions between waking and sleeping states^68^. Thus, a poor ipRGCs integration of the light signal, as we found in individuals with IH, could lead to a lower stimulation of the wake system and/or a lower inhibition of the brain structures involved in sleep induction. Taken all together, this may suggest a “light related vulnerability to sleepiness” in individuals suffering from IH.

The main limitation of this study is the relatively small sample size due to the rarity of IH, which may reduce statistical power, although studies working on this topic reported lower sample sizes. Nevertheless, it will be necessary in the future to confirm these results with a larger sample size, with inclusions in different sleep centers. Another limitation of this study is the lack of the recent light history that can alter the ipRGCs sensitivity and potentially influence our measures^38^. Indeed, it would be interesting in future studies to collect the light quantity received prior to the pupillometry protocol. However, the blue light exposure of 5 min was used in the current study to obtain a completed light adaptation of ipRGCs as mentioned earlier^38^. This long time of exposure, associated with a prior adaptation of 30 min to the light room, seems to be more appropriate than transient flash to reduce the effect of light history, and may have limit this bias.

To our knowledge, this is the first study to compare the PIPR between a population suffering from IH and healthy subjects. We observed that patients with IH had a lower PIPR than controls. These findings suggest specific changes of melanopsin-based photic regulation in IH, which has never been shown before. The melanopsin-specific PIPR may be an innovative trait marker of IH with long sleep time and the pupillometry could be a promising tool to better understand and characterize central hypersomnia. Further understanding of the melanopsin system in IH may improve treatment. Indeed, if the melanopsin system is “hypofunctional” in IH with long sleep time, natural light or artificial light therapy, with adapted parameters such as longer time of exposure, might be a promising innovative therapeutic approach to improve wakefulness. In addition, the PIPR might also help predicting which individuals are responders or not to treatments in this population. In the future, it will be necessary to compare our results with other groups of hypersomnia such as IH without prolonged total sleep time, narcolepsy type 1 and 2, and hypersomnia associated to MDD to further validate the specificity of this marker.

## Data Availability

The data that support the findings will be available in upon request from the corresponding author.

## References

[1] American Academy of Sleep Medicine (2014). International Classification of Sleep Disorders (ICSD-3).

[2] American Psychiatric Association. in *Diagnostic and Statistical Manual of Mental Disorders (DSM-5)*, 5th Edn. (American Psychatric Association, 2013).

[3] Ohayon MM, Reynolds CF, Dauvilliers Y (2013). Excessive sleep duration and quality of life: Excessive Sleep in USA. Ann. Neurol..

[4] Billiard M, Sonka K (2016). Idiopathic hypersomnia. Sleep Med. Rev..

[5] Ali M, Auger RR, Slocumb NL, Morgenthaler TI (2009). Idiopathic hypersomnia: Clinical features and response to treatment. J Clin Sleep Med.

[6] Vernet C, Arnulf I (2009). Idiopathic hypersomnia with and without long sleep time: A controlled series of 75 patients. Sleep.

[7] Lopez R, Barateau L, Evangelista E, Dauvilliers Y (2017). Depression and hypersomnia. Sleep Med. Clin..

[8] Landzberg D, Trotti LM (2019). Is idiopathic hypersomnia a circadian rhythm disorder?. Curr. Sleep Med. Rep..

[9] Geoffroy PA (2018). Insomnia and hypersomnia in major depressive episode: Prevalence, sociodemographic characteristics and psychiatric comorbidity in a population-based study. J. Affect. Disord..

[10] Lecendreux M (2015). Attention-deficit/hyperactivity disorder (ADHD) symptoms in pediatric narcolepsy: A cross-sectional study. Sleep.

[11] Lopez R (2020). Association of inattention, hyperactivity, and hypersomnolence in two clinic-based adult cohorts. J. Atten. Disord..

[12] Zele AJ, Adhikari P, Cao D, Feigl B (2019). Melanopsin and cone photoreceptor inputs to the afferent pupil light response. Front. Neurol..

[13] Dacey DM (2005). Melanopsin-expressing ganglion cells in primate retina signal colour and irradiance and project to the LGN. Nature.

[14] Altimus CM (2008). Rods-cones and melanopsin detect light and dark to modulate sleep independent of image formation. Proc. Natl. Acad. Sci..

[15] Hattar S (2002). Melanopsin-containing retinal ganglion cells: Architecture, projections, and intrinsic photosensitivity. Science.

[16] Vandewalle G, Maquet P, Dijk D-J (2009). Light as a modulator of cognitive brain function. Trends Cogn. Sci..

[17] Rüger M (2013). Human phase response curve to a single 6.5 h pulse of short-wavelength light: Blue light phase response curve. J. Physiol..

[18] Hubbard J (2021). Dissecting and modeling photic and melanopsin effects to predict sleep disturbances induced by irregular light exposure in mice. Proc. Natl. Acad. Sci. USA.

[19] An K (2020). A circadian rhythm-gated subcortical pathway for nighttime-light-induced depressive-like behaviors in mice. Nat. Neurosci..

[20] Zhang Z, Beier C, Weil T, Hattar S (2021). The retinal ipRGC-preoptic circuit mediates the acute effect of light on sleep. Nat. Commun..

[21] Schmidt TM (2011). Melanopsin-positive intrinsically photosensitive retinal ganglion cells: From form to function. J. Neurosci..

[22] Hatori M, Panda S (2010). The emerging roles of melanopsin in behavioral adaptation to light. Trends Mol. Med..

[23] van der Meijden WP (2015). Post-illumination pupil response after blue light: Reliability of optimized melanopsin-based phototransduction assessment. Exp. Eye Res..

[24] Bailes HJ, Lucas RJ (2013). Human melanopsin forms a pigment maximally sensitive to blue light (*λ*_max_ ≈ 479 nm) supporting activation of G_q/11_ and G_i/o_ signalling cascades. Proc. R. Soc. B.

[25] van der Meijden WP (2018). Sustained effects of prior red light on pupil diameter and vigilance during subsequent darkness. Proc. R. Soc. B.

[26] Gamlin PDR (2007). Human and macaque pupil responses driven by melanopsin-containing retinal ganglion cells. Vision. Res..

[27] Adhikari P, Zele AJ, Feigl B (2015). The post-illumination pupil response (PIPR). Invest. Ophthalmol. Vis. Sci..

[28] LeGates TA, Fernandez DC, Hattar S (2014). Light as a central modulator of circadian rhythms, sleep and affect. Nat. Rev. Neurosci..

[29] van der Meijden WP (2016). Individual differences in sleep timing relate to melanopsin-based phototransduction in healthy adolescents and young adults. Sleep.

[30] Laurenzo SA (2016). Pupillary response abnormalities in depressive disorders. Psychiatry Res..

[31] Berman G (2018). Decreased retinal sensitivity in depressive disorder: A controlled study. Acta Psychiatr. Scand..

[32] Abbott SM, Choi J, Wilson J, Zee PC (2021). Melanopsin-dependent phototransduction is impaired in delayed sleep–wake phase disorder and sighted non-24-hour sleep–wake rhythm disorder. Sleep.

[33] Lecrubier Y (1997). The Mini International Neuropsychiatric Interview (MINI). A short diagnostic structured interview: Reliability and validity according to the CIDI. Eur. Psychiatr..

[34] Carskadon MA (1986). Guidelines for the multiple sleep latency test (MSLT): A standard measure of sleepiness. Sleep.

[35] Littner MR (2005). Practice parameters for clinical use of the multiple sleep latency test and the maintenance of wakefulness test. Sleep.

[36] Zele AJ, Feigl B, Smith SS, Markwell EL (2011). The circadian response of intrinsically photosensitive retinal ganglion cells. PLoS ONE.

[37] Mure LS, Rieux C, Hattar S, Cooper HM (2007). Melanopsin-dependent nonvisual responses: Evidence for photopigment bistability in vivo. J. Biol. Rhythms.

[38] Wong KY, Dunn FA, Berson DM (2005). Photoreceptor adaptation in intrinsically photosensitive retinal ganglion cells. Neuron.

[39] Kankipati L, Girkin CA, Gamlin PD (2010). Post-illumination pupil response in subjects without ocular disease. Invest. Ophthalmol. Vis. Sci..

[40] Kelbsch C (2019). Standards in pupillography. Front. Neurol..

[41] Horne JA, Östberg O (1977). Individual differences in human circadian rhythms. Biol. Psychol..

[42] Taillard J, Philip P, Chastang J-F, Bioulac B (2004). Validation of horne and ostberg morningness–eveningness questionnaire in a middle-aged population of French workers. J. Biol. Rhythms.

[43] Johns MW (1991). A new method for measuring daytime sleepiness: The Epworth Sleepiness Scale. Sleep.

[44] Kaminska M (2010). The Epworth Sleepiness Scale: Self-administration versus administration by the physician, and validation of a French version. Can. Respir. J..

[45] Buysse DJ, Reynolds CF, Monk TH, Berman SR, Kupfer DJ (1989). The Pittsburgh sleep quality index: A new instrument for psychiatric practice and research. Psychiatry Res..

[46] Ait-Aoudia M (2013). Validation of the French version of the Pittsburgh Sleep Quality Index Addendum for posttraumatic stress disorder. Eur. J. Psychotraumatol..

[47] Gardenas, J. *et al*. Echelles et outils d’évaluation en médecine générale. 54 (2002).

[48] Terman M, Williams JBW, White TM, Partonen T, Pandi-Perumal SR (2009). Assessment instruments. Seasonal Affective Disorder.

[49] Tekin K (2018). Static and dynamic pupillometry data of healthy individuals: Normative data for pupillometry. Clin. Exp. Optom..

[50] Arnulf I, Leu-Semenescu S, Dodet P (2019). Precision medicine for idiopathic hypersomnia. Sleep Med. Clin..

[51] Feigl B, Ojha G, Hides L, Zele AJ (2018). Melanopsin-driven pupil response and light exposure in non-seasonal major depressive disorder. Front. Neurol..

[52] Münch M, Kawasaki A (2013). Intrinsically photosensitive retinal ganglion cells: Classification, function and clinical implications. Curr. Opin. Neurol..

[53] Kollarits CR, Lechman J, Kollarits FJ, Gillin JC (1982). The pupil dark response in narcolepsy. Curr. Eye Res..

[54] McLaren JW, Hauri PJ, Lin S-C, Harris CD (2002). Pupillometry in clinically sleepy patients. Sleep Med..

[55] Merritt SL, Schnyders HC, Patel M, Basner RC, O’Neill W (2004). Pupil staging and EEG measurement of sleepiness. Int. J. Psychophysiol..

[56] Wilhelm H, Lüdtke H, Wilhelm B (1998). Pupillographic sleepiness testing in hypersomniacs and normals. Graefe’s Arch. Clin. Exp. Ophthalmol..

[57] Pressman MR (1984). Patterns of daytime sleepiness in narcoleptics and normals: A pupillometric study. Electroencephalogr. Clin. Neurophysiol..

[58] Bär K-J (2004). The influence of major depression and its treatment on heart rate variability and pupillary light reflex parameters. J. Affect. Disord..

[59] Larson MD, Behrends M (2015). Portable infrared pupillometry: A review. Anesth. Analg..

[60] Sforza E, Roche F, Barthélémy JC, Pichot V (2016). Diurnal and nocturnal cardiovascular variability and heart rate arousal response in idiopathic hypersomnia. Sleep Med..

[61] Cajochen C (2007). Alerting effects of light. Sleep Med. Rev..

[62] Comtet H (2019). Light therapy with boxes or glasses to counteract effects of acute sleep deprivation. Sci. Rep..

[63] Hubbard J, Ruppert E, Gropp C-M, Bourgin P (2013). Non-circadian direct effects of light on sleep and alertness: Lessons from transgenic mouse models. Sleep Med. Rev..

[64] Stothard ER (2017). Circadian entrainment to the natural light-dark cycle across seasons and the weekend. Curr. Biol..

[65] Viola AU, James LM, Schlangen LJ, Dijk D-J (2008). Blue-enriched white light in the workplace improves self-reported alertness, performance and sleep quality. Scand. J. Work Environ. Health.

[66] Wehr TA (1991). The durations of human melatonin secretion and sleep respond to changes in daylength (photoperiod). J. Clin. Endocrinol. Metab..

[67] Tsai JW (2009). Melanopsin as a sleep modulator: Circadian gating of the direct effects of light on sleep and altered sleep homeostasis in Opn4−/− mice. PLoS Biol..

[68] Cheng J (2020). The interaction between the ventrolateral preoptic nucleus and the tuberomammillary nucleus in regulating the sleep-wakefulness cycle. Front. Neurosci..

